# Dengue Virus Infection Alters Inter-Endothelial Junctions and Promotes Endothelial–Mesenchymal-Transition-like Changes in Human Microvascular Endothelial Cells

**DOI:** 10.3390/v15071437

**Published:** 2023-06-26

**Authors:** Manuela Escudero-Flórez, David Torres-Hoyos, Yaneth Miranda-Brand, Ryan L. Boudreau, Juan Carlos Gallego-Gómez, Miguel Vicente-Manzanares

**Affiliations:** 1 Molecular and Translation Medicine Group, University of Antioquia, Medellin 050010, Colombia; manuela.escuderof@udea.edu.co (M.E.-F.); david.torresh@udea.edu.co (D.T.-H.); yaneth.miranda@udea.edu.co (Y.M.-B.); 2 Division of Cardiovascular Medicine, Department of Internal Medicine, University of Iowa Carver College of Medicine, Iowa City, IA 52242, USA; ryan-boudreau@uiowa.edu; 3 Molecular Mechanisms Program, Centro de Investigación del Cáncer, Instituto de Biología Molecular y Celular del Cáncer, Consejo Superior de Investigaciones Científicas (CSIC), Universidad de Salamanca, 37007 Salamanca, Spain

**Keywords:** dengue virus, endothelial cells, cell migration, c-ABL, EMT, EndMT

## Abstract

Dengue virus (DENV) is a pathogenic arbovirus that causes human disease. The most severe stage of the disease (severe dengue) is characterized by vascular leakage, hypovolemic shock, and organ failure. Endothelial dysfunction underlies these phenomena, but the causal mechanisms of endothelial dysfunction are poorly characterized. This study investigated the role of c-ABL kinase in DENV-induced endothelial dysfunction. Silencing c-ABL with artificial miRNA or targeting its catalytic activity with imatinib revealed that c-ABL is required for the early steps of DENV infection. DENV-2 infection and conditioned media from DENV-infected cells increased endothelial expression of c-ABL and CRKII phosphorylation, promoted expression of mesenchymal markers, e.g., vimentin and N-cadherin, and decreased the levels of endothelial-specific proteins, e.g., VE-cadherin and ZO-1. These effects were reverted by silencing or inhibiting c-ABL. As part of the acquisition of a mesenchymal phenotype, DENV infection and treatment with conditioned media from DENV-infected cells increased endothelial cell motility in a c-ABL-dependent manner. In conclusion, DENV infection promotes a c-ABL-dependent endothelial phenotypic change that leads to the loss of intercellular junctions and acquisition of motility.

## 1. Introduction

Dengue virus (DENV) is responsible for up to 100 million symptomatic infections per year worldwide [1,2]. Clinical presentation of infected patients includes several phases and degrees of severity. The most severe, life-threatening stage of the disease is known as severe dengue. One of its main features is a dramatic increase in vascular permeability and plasma extravasation that can cause the death of the patient if not detected in time. Classically, this phenomenon has been examined from an immunopathogenic perspective. DENV triggers the production of many non-neutralizing antibodies that enhance infection upon secondary exposure to non-identical virus strains [3]. DENV infection also triggers the release of cytokines and proinflammatory mediators that may alter endothelial integrity [4]. This is strikingly similar to the adverse effects of some anti-cancer therapies and septic shock [5].

An emerging hypothesis in the field is that severe dengue is caused by endothelial dysfunction. The barrier function of endothelial monolayers is linked to the dynamics of the cytoskeleton and diverse signaling pathways, including those controlling the integrity of cell–cell junctions and cellular contraction [6,7]. While endothelial cells are not the main target of DENV [8], infection of multiple cell types induces the release of soluble factors, including proinflammatory cytokines such as TNF-α, IL-1β, or IL-6 [4], and others such as FGF-2, TGF-α, GM-CSF, and IL-8 [9]. TNF-α and IL-1β induce vascular permeability [10], whereas some of the latter induce endothelial cell migration. In general, motility of monolayer-forming cells requires the weakening of adherens and tight junctions [9]. Several reports have shown that virus components such as the non-structural protein 1 (NS1) trigger vascular leakage [11,12]. Therefore, the combination of viral products and inflammatory mediators compromise the barrier function of endothelial monolayers throughout the host, constituting a hallmark of severe dengue.

Enhanced endothelial permeability is usually due to a combination of strong contraction and weakening of cell–cell junctions. Inward contraction of the actin cytoskeleton applies tension to cell–cell junctions, mechanically disrupting cellular monolayers. DENV-induced extracellular mediators trigger signaling pathways that enhance contraction, thereby potentially weakening cell–cell junctions. Contraction is usually mediated by the activation of Rho-GTPase-dependent responses that converge on myosin II activation [13], whereas weakening of cell–cell junctions may involve the transcriptional or translational repression of proteins that form homotypic interactions between neighbor cells, e.g., cadherins [14]. Contraction and transcriptional alterations leading to increased cell motility often involve the activity of non-receptor Tyr kinases. Abelson (c-ABL) kinase is a Src-related kinase that drives oncogenesis in leukemias positive for the Philadelphia chromosome. In these disorders, c-ABL fusion to the Break Cluster Region (BCR-ABL) causes the ectopic activation of the fusion product, generating c-ABL-dependent survival signals [15]. In a non-leukemogenic, physiological context, wild type c-ABL is involved in different signal pathways that control cell morphology [16,17] as well as inter-endothelial junctions [18]. c-ABL has a context-specific, regulatory role on cell–cell junctions. In normal cells, e.g., mouse embryo fibroblasts, c-ABL is required for the preservation of the integrity of cell–cell junctions [18]. Conversely, in colon cancer cells, c-ABL is required for PDGF-triggered EMT [19]. In this regard, c-ABL has been linked to cellular trans-differentiation processes such as Endothelial–Mesenchymal Transition (EndMT) [20]. VEGF-dependent stimulation of endothelial cells triggers the activation of c-ABL [21], increasing contractility and driving VE-cadherin internalization from adherens junctions into the cytosol [22]. TGF-β also activates c-ABL, favoring the expression of transcription factors such as SNAIL and TWIST and other mesenchymal markers, and repressing the expression of endothelial-specific proteins, thereby promoting cell scattering and motility [23]. These data indicate that c-ABL is a crucial checkpoint that is involved in the regulation of cell contraction and controls the decrease in expression of proteins involved in cell–cell contacts that favors motility, thereby compromising the integrity of endothelial cell junctions.

Previously, potential antiviral effects have been reported for c-ABL kinase inhibitors. Pretreatment of epithelial cells with GNF-2 (a specific, allosteric c-ABL inhibitor) significantly reduced viral entry via clathrin-mediated endocytosis and interfered with viral replication [24]. However, the possible antiviral effects of c-ABL inhibition have been poorly studied in endothelial cells, which are key players in vascular leakage associated with severe dengue [25]. The hypothesis of the present work is that the mechanism by which severe dengue induces vascular leakage involves endothelial targeting of c-ABL. Such targeting either affects cell–cell junctions by increasing contraction and/or by triggering trans-differentiation phenomena that compromises the barrier function of endothelial monolayers through the weakening of cell–cell junctions.

In this study, DENV2 infection or incubation of endothelial cells with conditioned media from infected cells, increased expression of the cellular kinase c-ABL, which in turn elevated the levels of CRKII phosphorylation. Both treatments also decreased the expression of junctional proteins VE-cadherin and ZO-1 and increased motility as well as the expression of mesenchymal markers N-cadherin and the intermediate filament vimentin. Importantly, pharmacological inhibition of c-ABL with imatinib or silencing c-ABL expression attenuated these phenotypic changes. Taken together, these data not only postulate c-ABL as a possible therapeutic target for DENV-induced vascular leakage but also point to the existence of c-ABL-dependent trans-differentiation events that potentially mediate DENV infection and the pathogenesis of severe dengue.

## 2. Materials and Methods

### 2.1. Reagents

Imatinib mesylate inhibitor (STI571) was purchased from SelleckChem. Stock solutions were prepared in sterile Milli-Q water and stored at −20 °C until use. Roswell Park Memorial Institute Medium (RPMI), Dulbecco’s Modified Eagle’s Medium (DMEM), 3-(4,5-dimethylthiazol-2-yl)-2,5-diphenyl tetrazolium bromide (MTT), XtremeGENE™ HP DNA Transfection Reagent, and FluorSave (Calbiochem) were supplied by Merck. Fetal bovine serum (FBS) and tissue culture antibiotic cocktail Penicillin/Streptomycin (PS) were provided by Invitrogen.

Anti-c-ABL (rabbit polyclonal IgG) was purchased from Santa Cruz Biotechnology. The anti-CRKII (rabbit polyclonal p-Tyr221) antibody was provided by LSBio. Anti-wrap (mouse monoclonal flavivirus E-glycoprotein) and anti-vimentin (mouse monoclonal) antibodies were supplied by ABCAM. The anti-VE-cadherin antibody (mouse monoclonal CD144) was purchased from Merck. Anti-ZO-1 (Rabbit Polyclonal), anti-N-cadherin (mouse monoclonal), anti-α/β-tubulin (mouse monoclonal), and secondary (Alexa Fluor 488 and Alexa Fluor 594) antibodies, Alexa Fluor 594-conjugated phalloidin mycotoxin, and Hoechst probe were supplied by Invitrogen. IRDye 680LT (goat anti-rabbit) and IRDye 800CW (goat anti-mouse) antibodies were provided by LI-COR Biosciences.

### 2.2. Virus and Cell Lines

Dengue virus type 2 (DENV-2, New Guinea strain), originally donated by María Elena Peñaranda and Eva Harris (University of California, Berkeley) was amplified in C6/36 HT cells (*Aedes albopictus*-ATCC^®®^ CRL-1660™) and titrated in BHK-21 cells (hamster kidney cells, ATCC^®®^ CCL-10™). HMEC-1 cells (human dermal microvascular endothelial cells, ATCC^®®^ CRL-3243™) were maintained in basal growth medium (10% FBS RPMI supplemented with 10 mM L-glutamine, 100 units/mL penicillin/streptomycin (P/S), 10 ng/mL epidermal growth factor (hEGF), 1 µg/mL hydrocortisone) and incubated at 37 °C in a 5% CO_2_, humidified atmosphere. BHK-21 cells were maintained in 10% FBS DMEM supplemented with 100 U/mL penicillin/streptomycin, and 10 mM L-Glutamine incubated at 37 °C and 5% CO_2_ and C6/36 HT cells with 10% FBS L-15 medium and 100 U/mL P/S at 34 °C and 5% CO_2_.

### 2.3. Plasmid Transfection

HMEC-1 cells were transfected with the plasmid pFBAAVmU6miABL1 CMVeGFP SV40pA (containing the microRNA sequence targeting c-ABL and GFP) or the same plasmid containing a scrambled version of the targeting sequence (pFBAAVmU6miScramble CMVeGFPSV40pA). Both plasmids were kindly donated by Dr. Ryan Boudreau (Gene Transfer Vector Core, Carver College of Medicine, University of Iowa, USA). Transfections were performed using XtremeGENE™ HP DNA Transfection Reagent and optimal conditions for these assays were standardized using a 2:1 XtremeGENE™–DNA ratio.

### 2.4. Amplification and Virus Titration

For viral replication and amplification, C6/36 HT cells were seeded in L-15 medium supplemented with 10% FBS and 100 U/mL P/S in 75 cm^2^ flasks at 70% confluence, infected with DENV-2 at a Multiplicity of Infection (MOI) of 0.01 PFU/mL in a final volume of 1 mL and incubated at 34 °C in a 5% CO_2_ atmosphere for two hours. Subsequently, 6 mL of L-15 with 2% FBS was added at the end of the incubation time without removing the viral inoculum. After 6 days of infection and confirming the occurrence of syncytia in ≥50% of the cell monolayer (using an Olympus IX-81 microscope), the supernatants were collected and stored at −70 °C until subsequent quantification. The Plaque Unit Formation assay was used to determine DENV titer. Briefly, 7.5 × 10^4^ BHK-21 cells were seeded in 24-well plates in DMEM supplemented with 2% FBS. The following day, dilutions from the virus to be titrated were added to cells in triplicate. Cells were incubated at 37 °C under 5% CO_2_ atmosphere for 2 h. The viral inoculum was then discarded from all infected wells and carboxymethylcellulose (CMC) with a viscosity of 1.5%. was added to each well. On the sixth day, the cells were fixed with a solution containing 3.5% PFA and 0.2% formaldehyde–crystal violet, and plaque forming units (PFUs) were quantified. The data are expressed as PFU/mL.

### 2.5. Infection of Microvascular Endothelial Cells and Production of Conditioned Media

The optimal MOI for HMEC-1 infection was determined by the plaque-forming unit assay. Briefly, triplicates of 1.8 × 10^5^ HMEC-1 cells were seeded in 24-well dishes in RPMI supplemented with 10% FBS. They were incubated at 37 °C and 5% CO_2_ for 24 h to allow adherence, washed with PBS, and infected with DENV-2 at a MOI of 5 (PFU/cell) for two hours. After this time, the inoculum was removed, the cells were washed with PBS and 2% FBS RPMI was added and left to incubate for 24, 48, 72, 96, and 120 h. At each time point, supernatants were collected and stored at −80 °C for subsequent titration. Viral titer was quantified as described in Section 2.4. Unless indicated otherwise, a MOI of 5 (PFUs/cell) was used throughout the study. Conditioned media were collected from mock- or DENV-infected HMEC-1 cells at 24, 48, 72, 96, and 120 hpi (hours post-infection). Virus inactivation was carried out by UVC (254 nm) irradiation for 30 min and tested in BHK-21 cells as described in Section 2.4.

### 2.6. Early-Stage and Post-Infective Stage Inhibition Assays

For evaluation of viral entry, imatinib (3 and 6 µM) was added to 80% confluent cell monolayers and incubated for 1 h at 37 °C in a 5% CO_2_ atmosphere. The cells were subsequently washed with PBS and inoculated with a MOI of 5 (PFU/cell) for 2 h at 37 °C and 5% CO_2_. Subsequently, the infectious medium was removed, the cells were washed with PBS, and 2% FBS RPMI was added and incubated for 72 h at 37 °C and 5% CO_2_. The supernatants were collected at the indicated time and stored at −80 °C until titration.

For the post-infective stage assay, the cell monolayer at 80% confluence was infected with a MOI of 5 (PFU/cell) for 2 h at 37 °C and 5% CO_2_. Afterward, the inoculum was removed, and the monolayer was washed with pre-warmed PBS. Next, the different concentrations of imatinib prepared in RPMI1640 containing 2% FBS were added and incubated again at 37 °C and 5% CO_2_ for 3 days. The supernatants from both experiments were collected at the indicated times and stored at −80 °C until titration.

### 2.7. Evaluation of Transfection Efficiency, c-ABL Expression Level, and CRKII Phosphorylation

For measurement of transfection efficiency, 1.8 × 10^5^ HMEC-1 cells were seeded on coverslips in RPMI supplemented with 10% FBS and transfected when they reached 80% confluency as described in Section 2.4. Cells were fixed at 24-, 48- and 72 h post-transfection with 3.8% PFA in CBS (Cytoskeletal Buffer with Sucrose, as described elsewhere [26]) for 20 min at 37 °C, treated with NH_4_Cl (50 mM), and blocked for 1 h using 5% fetal bovine serum in PBS. Cells were subsequently incubated with Hoescht 33,258 (nuclei labeling; 1:10,000) for 45 min, washed three times with PBS and mounted on slides. GFP fluorescence was observed using a fluorescence microscope (Olympus IX-81 microscope) fitted with a Hg lamp and appropriate filters using a 20× objective. Images were captured with a Nikon DS Camera DS-5M and quantified using ImageJ software to determine the percentage of GFP-positive cells.

The relative expression level of c-ABL and CRKII phosphorylation were determined at 24, 48, and 72 h post-transfection in 96-well plates using In-Cell Western (ICW). Briefly, the cell monolayer was fixed (3.8% PFA in CBS), permeabilized (Triton X-100), blocked (5% bovine serum albumin), labeled for c-ABL (anti-c-ABL; 1:500 dilution) or phospho-CRKII (anti-p-Tyr221 CRKII; 1:500 dilution), and revealed with the antibody infra-red IRDye 800LT length (1:1000). In parallel, tubulin labeling was performed (mouse anti-α/β-tubulin; 1:500) and IRDye 800CW (1:1000 dilution) to use as a normalizing protein. Detection was performed with an Odyssey (LI-COR Biosciences). The results shown are the average of three independent assays with four technical replicates.

### 2.8. Determination of Actin Reorganization and Expression of VE-Cadherin, ZO-1, N-Cadherin, and Vimentin

Reorganization of the actin cytoskeleton and expression of endothelial and mesenchymal markers was observed by fluorescence microscopy (Olympus IX-81 microscope). For this purpose, 1.8 × 10^5^ HMEC-1 cells were seeded on coverslips in RPMI supplemented with 10% FBS. The next day, 80% confluent cells were transfected and/or infected with DENV-2 at a MOI of 5; or treated with culture supernatants from infected cells. After the treatments, cells were fixed using 3.8% PFA in CBS for 20 min, treated with NH_4_Cl (50 mM), permeabilized (Triton X-100), and blocked for 1 h (5% fetal bovine serum in 1× PBS). Subsequently, the cells were labeled with anti-VE-cadherin (mouse monoclonal CD144; 1:500), anti-N-cadherin (mouse monoclonal; 1:500), anti-ZO-1 (mouse monoclonal; 1:500), or anti-vimentin (mouse monoclonal; 1:500) for 1 h. After extensive rinsing with PBS, the cells were incubated with the appropriate species-specific secondary antibodies coupled to AlexaFluor488/594 and/or Alexa Fluor 594-conjugated phalloidin (1:1000) and Hoescht 33,258 (1:10,000) for 45 min, washed three times with PBS, and mounted on slides. GFP/Actin images were captured using an Olympus^®®^IX-81 Epifluorescence Microscope (Tokyo, Japan) equipped with a 60× objective, NA 1.40, coupled to an Olympus CCD DP30BW camera; the images were captured using Media Cybernetics, ImageProPlus^®®^ software (v.7), and processed using ImageJ (v. 1.53t). Staining images in Figures 5 and 6 were imaged with the same microscope using a 40× objective and processed and quantified (15 images per condition) in ImageJ.

### 2.9. Cell Migration Assays

To determine the migration efficiency of HMEC-1 cells in each treatment condition, 2 × 10^5^ cells were seeded in glass-bottomed Petri dishes (35 mm). The cells were incubated for 2 days until the formation of a cell monolayer and minutes before recording, the monolayer was wounded using an insulin (30 G) syringe. The cells were then treated with conditioned media from the infected (DENV2-CM) or control (MOCK-CM) cells combined with serum-free recording medium, in a ratio of 80/20. Imatinib was added at a final concentration of 6 µM. Where indicated, cells were previously transfected with amiScr or amic-ABL and then treated with DENV-CM.

Live cell frames were obtained at 40× magnification with a spinning disk confocal microscope (Olympus^®®^IX-81 DSU), coupled to Tokai-Hit Co^®®^ incubation and a gas mixing system (Shizuoka, Japan), equipped with an automated scanning system (PriorScientific) and autofocusing system (Laser Autofocus System). Frames were captured using an ultra-cooled OrcaR2CCD camera (Hamamatsu^®®,^ Shizuoka, Japan) with an electric multiplier, coupled to illumination systems with 150 W arc burners consisting of mercury–xenon or xenon lamps (Olympus^®®^-MT10 Illumination System). The system was controlled using Xcellence-Pro Software (Olympus^®®^).

Debugging and analysis from the videos consisted of extracting frames using the Windows media program VLC, and then the images were semi-assisted and segmented using the BioEdIP software (v7.0), which yields the cell-free area data in both percentage and number of pixels. The data were expressed as Percent Wound Area (%WAH) as a function of recording time, and as Percent Wound Reduction Effectiveness (%WR Effectiveness) as a function of each condition; 12 frames per video, representative of each hour of recording, were evaluated.

### 2.10. Statistical Analysis

IC_50_ values were calculated using the linear regression model in GraphPad Prism software. To compare viral titers (PFU/mL) and MFI of viral and endothelial components, the unpaired Student’s *t*-test was used. Protein expression level data obtained by In-Cell Western were analyzed by multiple Student’s *t*-tests. Finally, to assess whether there were significant differences in percent wound reduction effectiveness (%WRH effectiveness) and percent wound area (%WAH) between conditions, one-way ANOVA and the nonparametric Kruskal–Wallis’ test were applied, respectively. Multiple comparisons between groups were performed by Dunnett’s test in R software.

## 3. Results

### 3.1. DENV-2 Infection Increases the Expression of c-ABL Kinase and Its Phosphorylated Cellular Target CRKII in HMEC-1 Cells

**Figure 1:**
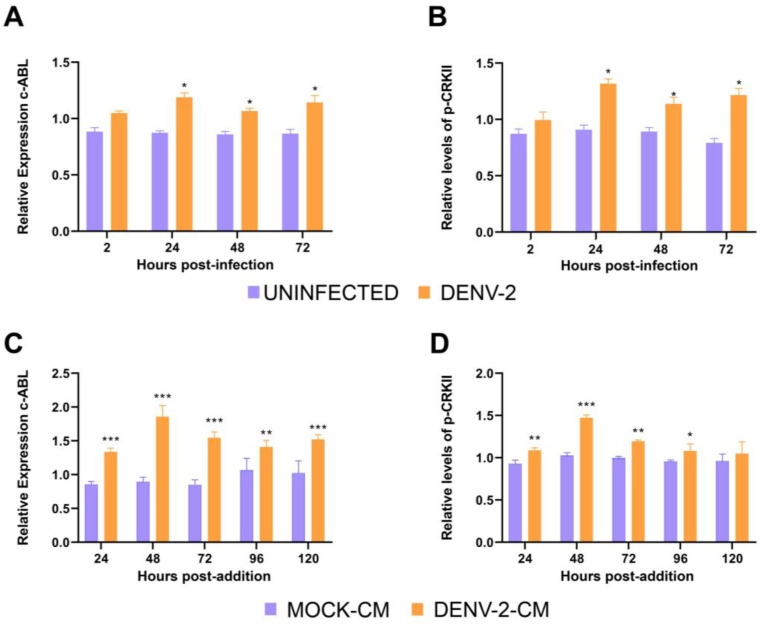
DENV-2 infection of HMEC-1 cells induces the release of soluble factors that promote c-ABL activity. (**A**) Relative expression of c-ABL kinase and (**B**) CRKII phosphorylation in HMEC-1 cells infected with DENV-2 MOI of 5. (**C**) Relative expression of c-ABL kinase and (**D**) CRKII phosphorylation in HMEC-1 cells exposed to conditioned media from DENV-2-infected cells. Conditioned media were obtained at 24, 48, 72, 96, and 120 hpi (DENV2-CM) and from the uninfected control (MOCK-CM). HMEC-1 cells were treated for 24 h, after which time they were fixed, and expression was quantified by ICW. Bars represent the standard deviation of three independent experiments performed in quadruplicate. The *p*-value was determined by multiple Student *t*-tests. (***): *p* < 0.001; (**): *p* < 0.01; (*): *p* < 0.05.

Previous studies have indicated the role of ABL in DENV infection [24]. To investigate the role of c-ABL in DENV infection of endothelial cells, the effect of DENV infection on the levels and/or activity of cellular c-ABL was assessed in the endothelial cell line HMEC-1. HMEC-1 cells infected with DENV-2 at a MOI of 5 were probed for c-ABL expression and CRKII phosphorylation at 24, 48 and 72 hpi. CRKII was used as a readout of increased c-ABL activity since it is directly phosphorylated by c-ABL in several cellular contexts [27,28]. DENV-2 infection significantly increased the levels of c-ABL (Figure 1A) as well as the degree of CRKII phosphorylation (Figure 1B). To test whether this effect was due to the secretion of specific soluble factors or the physical event of cellular infection, HMEC-1 cells were infected with DENV-2 at a MOI of 5 and supernatants of the infected cells were collected 24, 48, 72, 96, and 120 hpi. Conditioned media from DENV-2-infected cells were exposed to UV light to inactivate the virions present in the medium. The conditioned media significantly increased the relative expression of c-ABL kinase (Figure 1C) and CRKII phosphorylation (Figure 1D) compared to conditioned media from the uninfected control (MOCK-CM). These results indicate that the secretion of specific soluble factors from the endothelial cells infected with DENV-2 increases the levels of c-ABL as well as its kinase activity on canonical c-ABL substrates such as CRKII.

### 3.2. Imatinib Inhibits the Early Stages of DENV-2 Infection in HMEC-1 Cells

**Figure 2:**
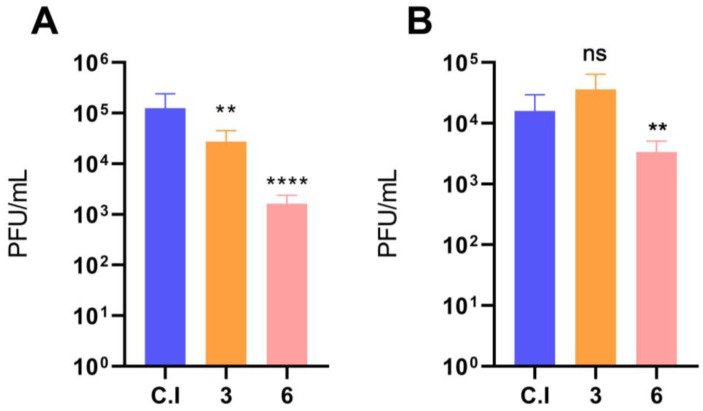
Antiviral activity of the pharmacological inhibitor imatinib in HMEC-1 cells during infection with DENV-2. (**A**) HMEC-1 cells were pretreated with the indicated doses of imatinib for 1 h. Cells were subsequently infected with DENV-2, and supernatants were collected at 72 h post-infection. (**B**) HMEC-1 cells that were previously infected were treated with imatinib until supernatants were collected 72 hpi. Viral titer was quantified by the plaque formation assay in BHK-21 cells. Bars represent the standard deviation of three independent experiments performed in duplicate. *p* value was determined by unpaired data Student’s *t*-test. (****): *p* value < 0.0001; (**): *p* < 0.01; ns: not significant.

The increase in expression of c-ABL upon DENV-2 infection suggested that its inhibition could block infection. Furthermore, an allosteric inhibitor of c-ABL, GNF-2, does inhibit DENV infection [24]. Thus, the possible inhibitory effect of the c-ABL inhibitor imatinib was evaluated in the initial stages of DENV-2 infection (viral entry) and post-infective stages (viral assembly and/or exit) in HMEC-1 cells. Thus, HMEC-1 cells were incubated before and after infection with two imatinib concentrations below the typical concentration used to inhibit c-ABL (10 µM, [5]). These doses were selected to prevent possible cytotoxic effects. Pretreatment of HMEC-1 cells with imatinib significantly reduced viral titer (Figure 2A). Conversely, cells treated with imatinib after DENV-2 infection displayed a less significant effect than pretreated cells, and only at the highest concentration tested (6 µM; Figure 2B).

### 3.3. c-ABL Depletion Reduces CRKII Phosphorylation and Promotes Actin Cytoskeleton Reorganization

**Figure 3:**
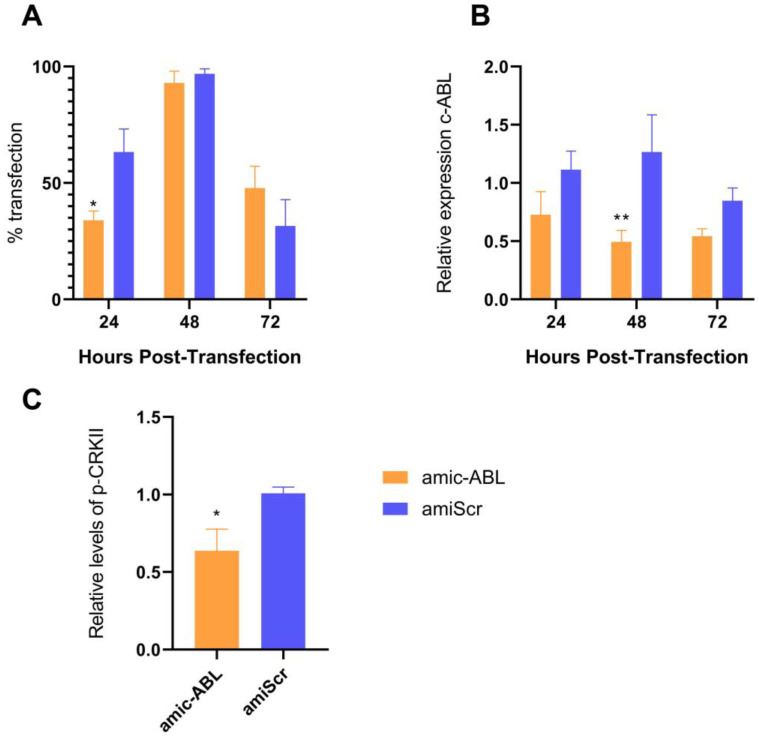
Depletion of c-ABL and reduction of CRK II phosphorylation in response to amiRNA-mediated silencing. (**A**) Transfection efficiency detected by expression of GFP. HMEC-1 cells were transfected with amic-ABL and amiScr, and GFP expression was observed at 24, 48, and 72 h post-transfection by fluorescence microscopy. Images were analyzed using ImageJ Software to quantify the number of GFP-positive cells over the total number of nuclei. (**B**) Relative expression of c-ABL kinase in HMEC-1 cells. Cells were transfected with amic-ABL and amiScr, fixed at 24, 48, and 72 h post-transfection, and levels of c-ABL measured by In-Cell Western (ICW). (**C**) Phosphorylation of CRKII. HMEC-1 cells were transfected with amic-ABL and amiScr, fixed at 48 h post-transfection, and phospho-CRKII was measured by ICW. Bars represent the standard deviation of three independent experiments performed in quadruplicate. Significance was determined using Student’s *t*-test. (**): *p* < 0.01; (*): *p* < 0.05.

**Figure 4:**
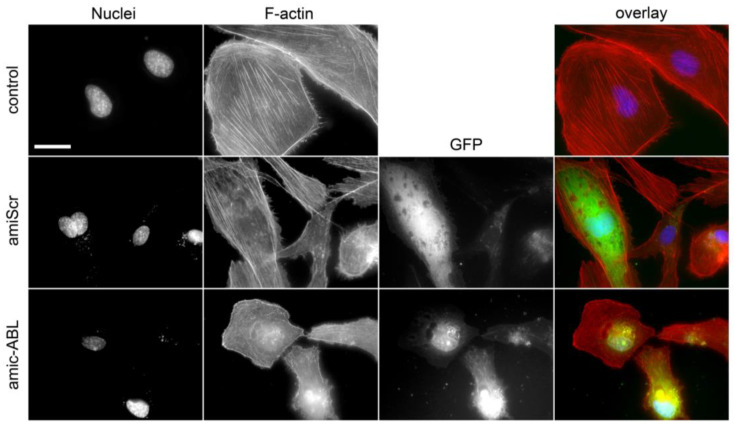
Silencing c-ABL induces actin cytoskeleton reorganization. HMEC-1 control, amiScr-, and amic-ABL-transfected cells were stained with phalloidin–AlexaFluor594. Actin stress fibers (in red) are readily observed in control and amiScr-transfected cells. In contrast, amic-ABL-transfected cells display a robust loss of stress fibers and increased presence of cortical F-actin. Expression of the GFP reporter gene (in green) is shown in the case of transfected cells. Scale bar = 20 µm.

To further evaluate the participation of the cellular kinase c-ABL in DENV-2 infection in the endothelial cell line HMEC-1, endogenous levels of c-ABL were reduced using a bicistronic plasmid containing the expression cassette of both the amiRNA [29] targeting the ABL1 gene under the murine U6 promoter and a GFP reporter under the CMV promoter (pFBAAVmU6miABL1-CMVeGFPSV40pA, referred to as amic-ABL throughout the text). Artificial microRNAs are superior to shRNA in that they promote less cytotoxicity and require lower levels for efficient downregulation. As a control, the same plasmid carrying a scrambled version of the ABL-targeting sequence (pFBAAVmU6miScramble-CMVeGFPSV40pA, noted as amiScr throughout the text) was used. This protocol yielded an approximately 90% transfection efficiency (according to the levels of GFP detected by fluorescence) at 48h and declining thereafter (Figure 3A). At this time point (48 h), an over 50% reduction in c-ABL expression was observed (Figure 3B). Consistent with the described role of c-ABL upstream of CRKII, a consistent decrease in phosphorylation of the latter was also observed (Figure 3C).

c-ABL is involved in the generation and maintenance of stress fibers through different mechanisms, e.g., activation of the RhoA/ROCK axis [30]. Consistent with this, depletion of c-ABL decreased the number of stress fibers in HMEC-1 cells (Figure 4).

### 3.4. DENV2 Infection Decreases VE-Cadherin and ZO-1 and Increases N-Cadherin and Vimentin in a c-ABL-Dependent Manner

**Figure 5:**
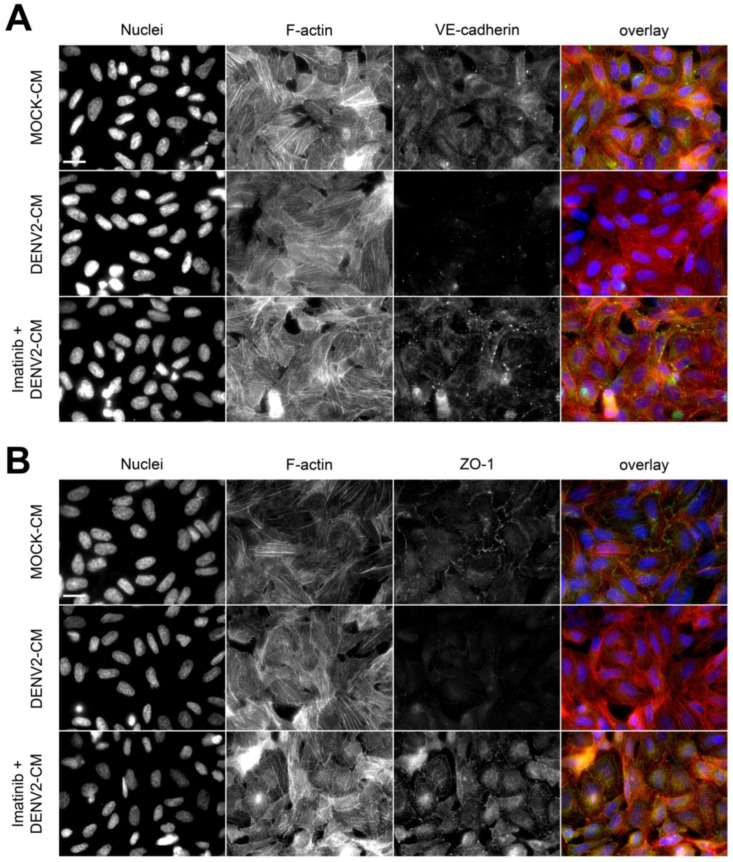

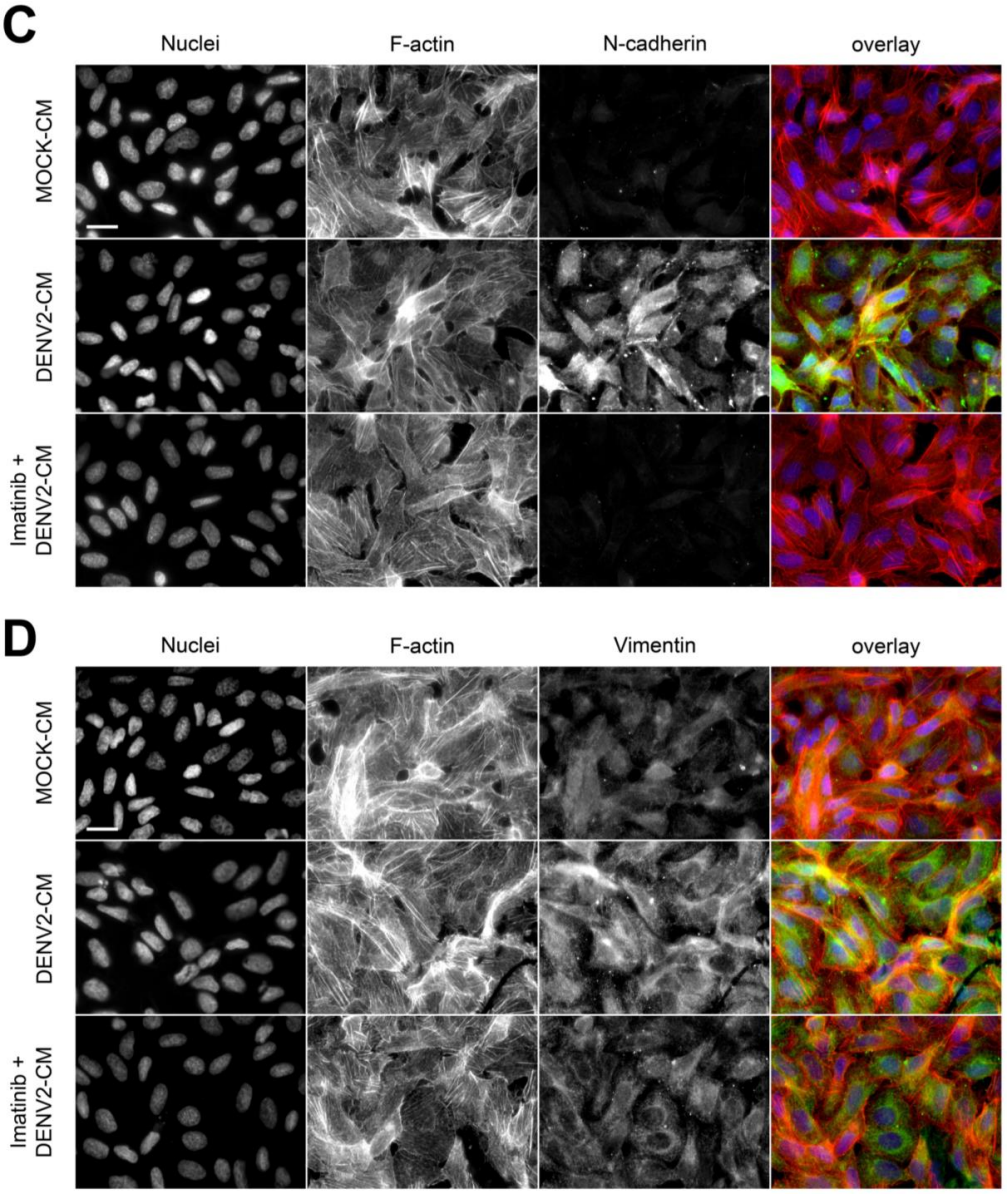

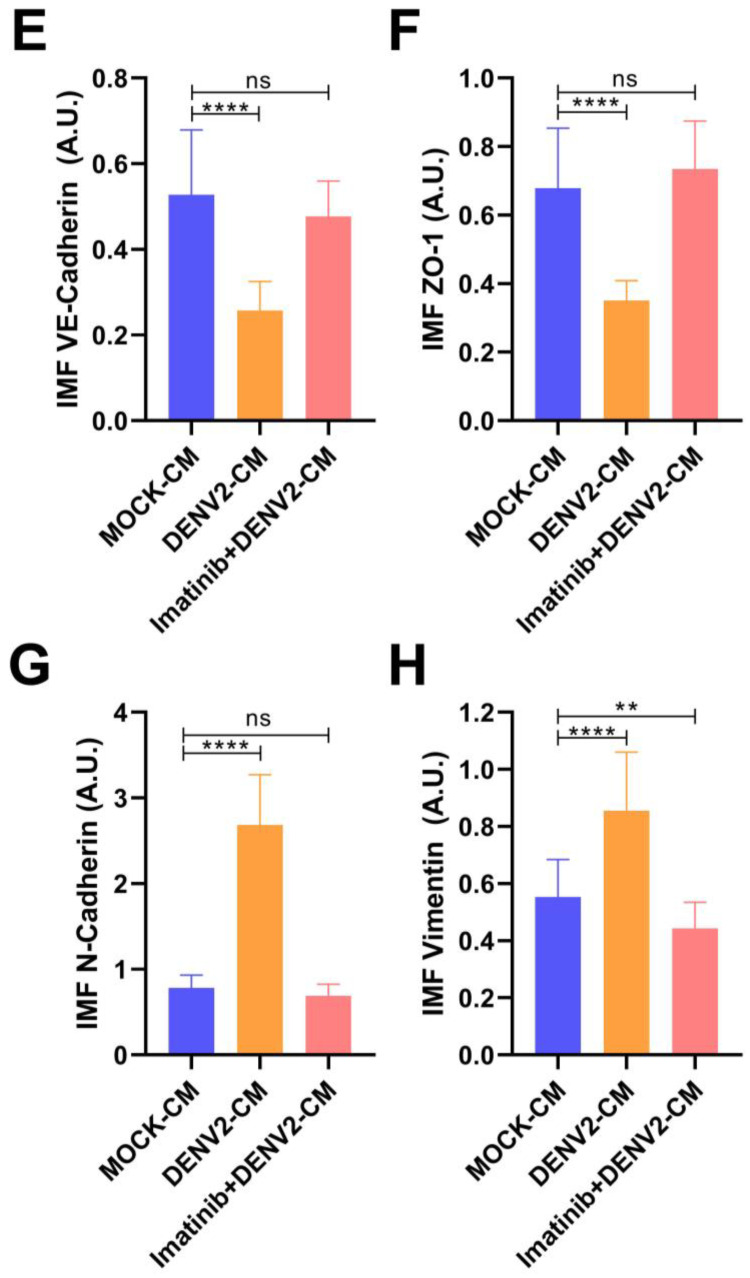
DENV2 infection reduces expression of VE-cadherin and ZO-1 and increased expression of N-cadherin and vimentin in HMEC-1 cells, which are reversed by treatment with imatinib. (**A**–**D**) Representative images of HMEC-1 cells treated with conditioned media collected from uninfected controls (MOCK-CM), DENV-2-infected cells (DENV2-CM), and DENV-2-infected cells plus imatinib (imatinib + DENV2-CM). Cells were stained for actin with phalloidin-AlexaFluor594 (in red), nuclei with Hoescht (in blue) and anti-VE-cadherin (**A**), anti-N-cadherin (**B**), anti-vimentin (**C**), or ZO-1 (**D**) (all in green). Scale bar = 20 µm. (**E**–**H**) Mean Fluorescence Intensity of the cellular components shown in (**A**–**D**) in HMEC-1 cells after the indicated treatments. MFI was quantified in 15 images per condition using ImageJ software from images acquired using the same microscopy settings. Bars represent the standard deviation of three independent experiments performed in duplicate. The *p*-value was determined by Student’s *t*-test for unpaired data. (****): *p* value < 0.0001; (**): *p* < 0.01; ns: not significant.

**Figure 6:**
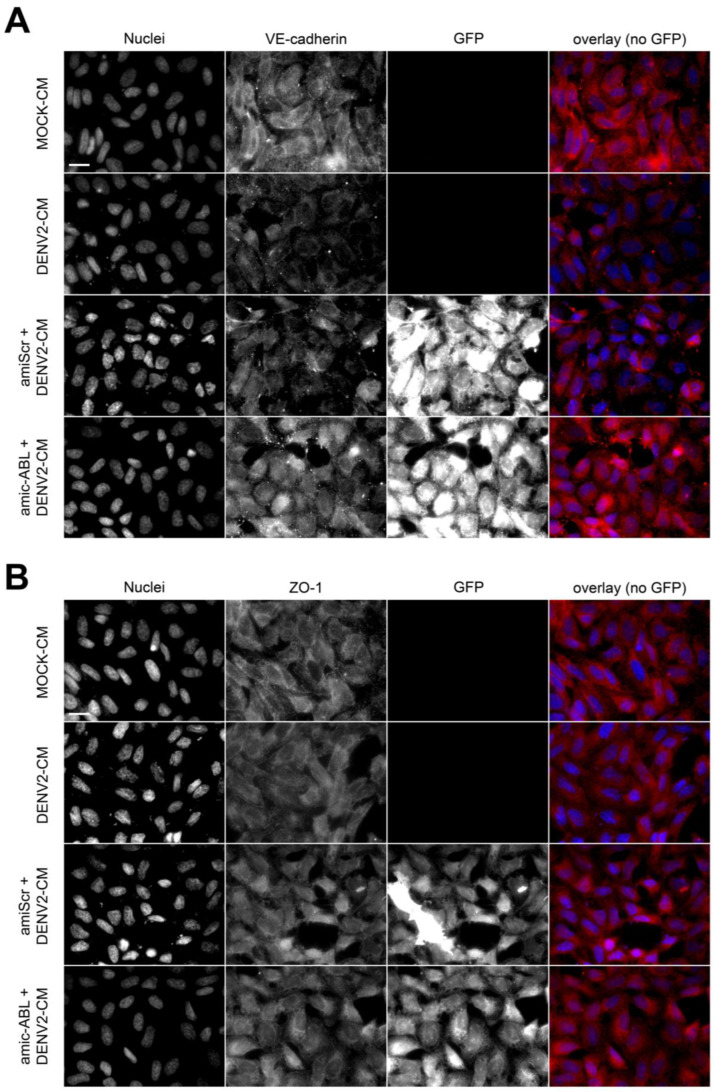

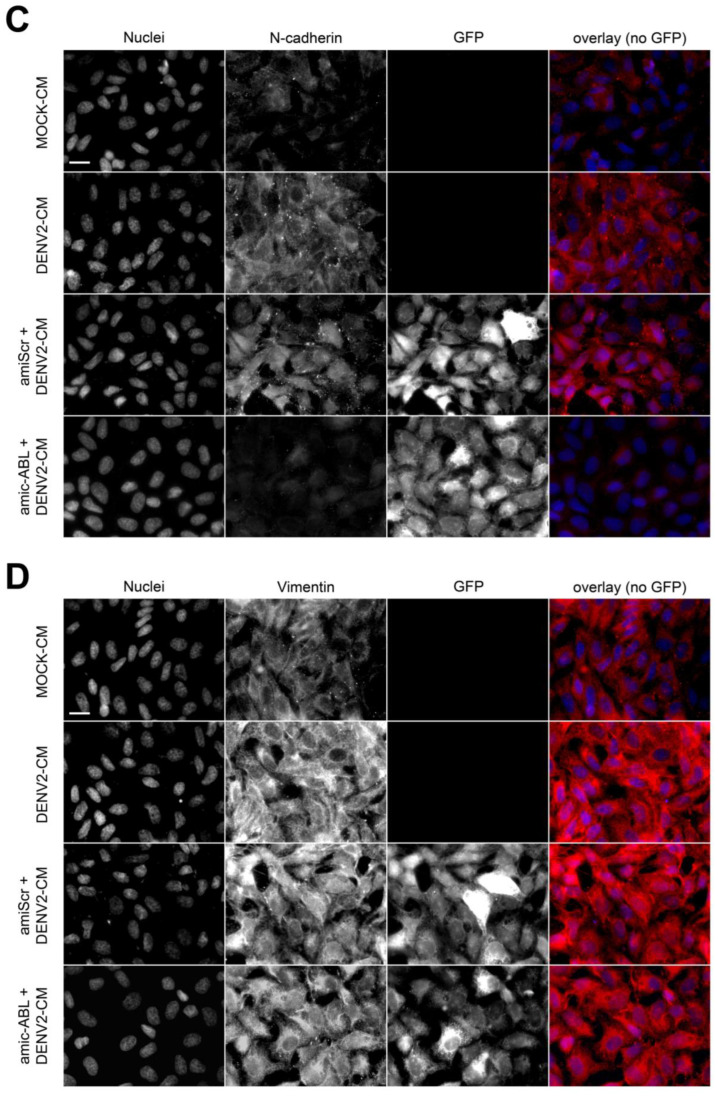

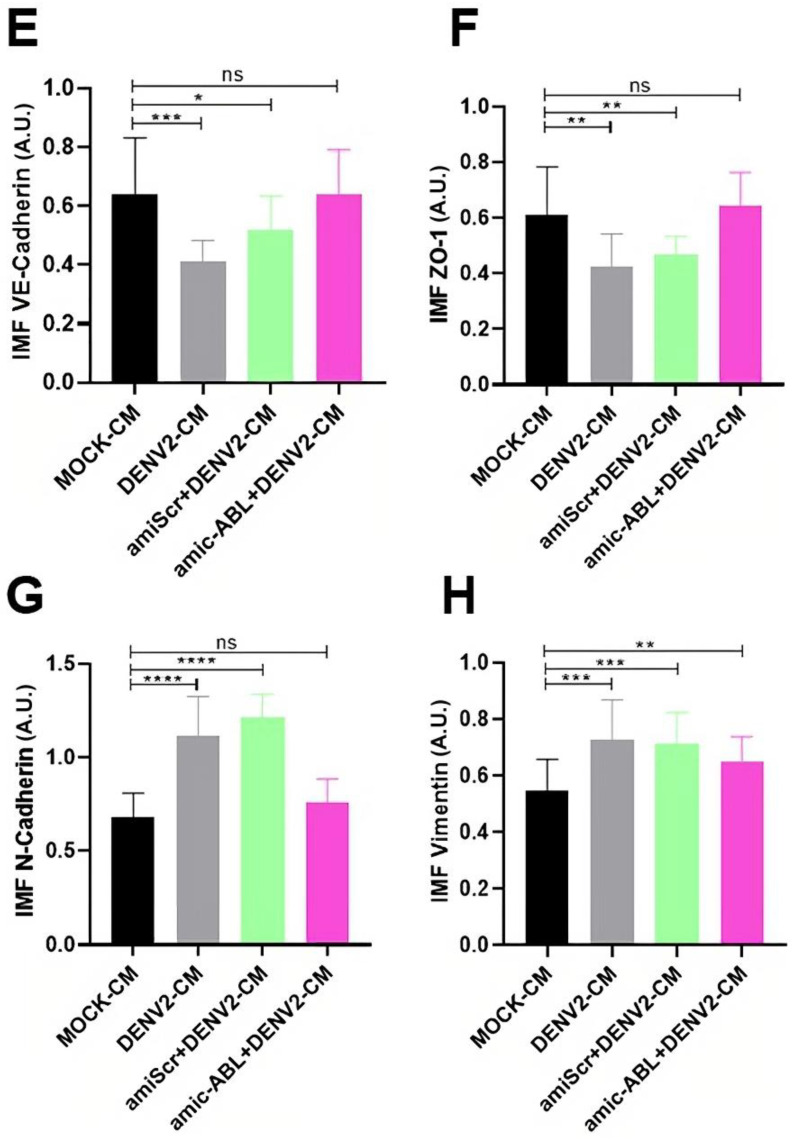
DENV2 infection reduced expression of VE-cadherin and ZO-1 and increased expression of N-cadherin and vimentin in HMEC-1 cells, and changes are attenuated by amiRNA-mediated depletion of c-ABL. (**A**–**D**) Representative images of HMEC-1 cells treated with conditioned media collected from uninfected control (MOCK-CM), DENV-2-infected cells (DENV2-CM), as well as HMEC-1 cells transfected with control (amiScr + DENV2-CM) or c-ABL-targeting plasmids (amic-ABL + DENV2-CM). Cells were stained for actin with phalloidin–AlexaFluor594 (in red), nuclei with Hoescht (in blue) and anti-VE-cadherin (**A**), ZO-1 (**B**), anti-N-cadherin (**C**), and anti-vimentin (**D**) (all in green). Scale bar = 20 µm. (**E**–**H**) Mean Fluorescence Intensity of the cellular components shown in (**A**–**D**) in HMEC-1 cells after the indicated treatments. MFI was quantified in 15 images per condition using ImageJ software from images acquired using the same microscopy settings. Bars represent the standard deviation of three independent experiments performed in duplicate. The *p*-value was determined by Student’s *t*-test for unpaired data. (****): *p* value < 0.0001; (***): *p* < 0.001; (**): *p* < 0.01; (*): *p* < 0.05; ns: not significant.

The formation of stable stress fibers is a hallmark of epithelial- and endothelial-to-mesenchymal transitions (EMT and EndMT, respectively), indicating a dominance switch from cell–cell-based to cell-matrix-based adhesion and the acquisition of motile capability [31]. Based on the inhibitory effect of imatinib on stress fiber formation (Figure 4) and the induction of c-ABL by DENV-2 infection (Figure 3), a hypothesis emerged according to which DENV infection could trigger EndMT in HMEC-1 cells in a c-ABL-dependent manner. To address this, expression of endothelial markers VE-cadherin and ZO-1 (markers of adherens and tight junctions, respectively) as well as mesenchymal markers N-cadherin and vimentin was assessed during infection. HMEC-1 cells were treated with conditioned media from uninfected control cells (MOCK-CM) and infected cells (DENV2-CM). Also, HMEC-1 cells treated with supernatants from infected cells were pre-treated with imatinib (imatinib + DENV2-CM). DENV2-CM induced a drastic reduction in the expression of VE-cadherin (Figure 5A,E) and ZO-1 (Figure 5B,F). Pretreatment of the cells with imatinib reversed this effect, preventing the DENV2-CM-induced decrease in the levels of VE-cadherin and ZO-1 (Figure 5A,B,E,F). DENV2-CM also increased the expression of N-cadherin and vimentin (Figure 5C,D,G,H). Again, pre-treatment with imatinib curbed the increased expression of N-cadherin and vimentin induced by conditioned media collected after DENV-2 infection (Figure 5G,H). Even though pre-treatment with imatinib decreased the apparent number of stress fibers independent of the presence of DENV2-CM (Figure 4), incubation with DENV2-CM alone had no apparent effect on them (Figure 5A–D). These data were confirmed independently using amiRNA-mediated silencing (Figure 6). Indeed, c-ABL depletion restored the levels of VE-cadherin (Figure 6A,E) and ZO-1 (Figure 6B,F), whereas it decreased the induction of N-cadherin (Figure 6C,G) and vimentin (Figure 6D,H). Similar effects were observed in DENV2-infected HMEC-1 cells (not shown). Together, these data are consistent with DENV2 infection triggering a cellular transition similar to EndMT that requires the normal functioning of c-ABL.

### 3.5. DENV-2 Infection Induces Cell Motility in a c-ABL-Dependent Manner

**Figure 7:**
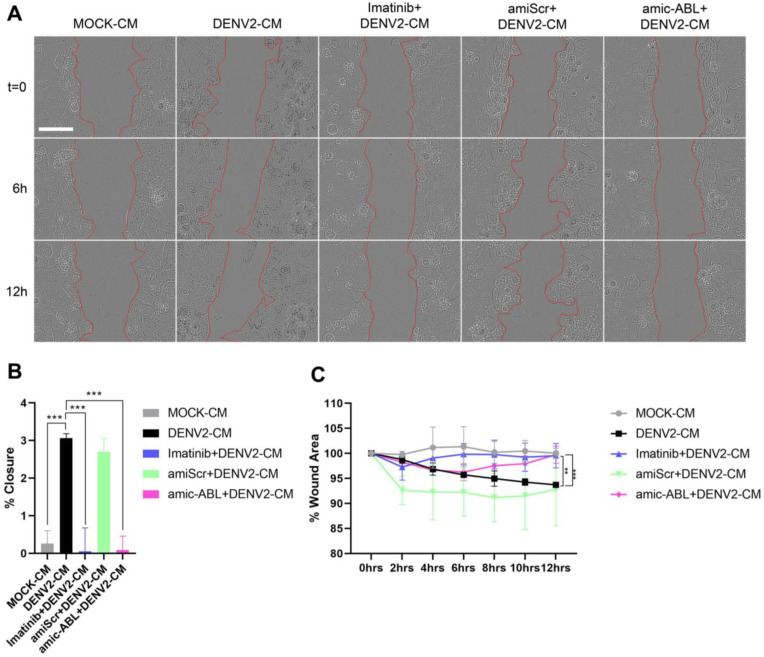
DENV-2 infection increases HMEC-1 wound healing migration in a c-ABL-dependent manner. (**A**) Representative frames of the indicated time points corresponding to wound healing experiments in HMEC-1 cells treated with control medium (MOCK-CM), conditioned medium from DENV-2-infected cells (DENV-2-CM), treated with imatinib (imatinib + DENV-2-CM) with depletion (amic-ABL + DENV-2-CM) or without depletion (amiScr + DENV-2-CM) of c-ABL in serum-free media. Images were captured as indicated in the Materials and Methods and segmented using BioEdIP software, which automatically delimits the cell-free area (boundaries shown in red). (**B**) Quantification of wound reduction. The difference between the initial %AH and final %AH was measured to determine the wound reduction effectiveness by condition. (**C**) Percentage of wound area (%AH) as a function of time in HMEC-1 cells by treatment. Bars represent the standard deviation of three independent experiments. The *p*-value was determined by a one-way ANOVA test for RH Effectiveness (%) and Kruskal–Wallis for %AH in R Software. (***): *p* < 0.001; (**): *p* < 0.01.

To further evaluate the effect of DENV-2 infection in the trans-differentiation of endothelium into mesenchymal-like cells, HMEC-1 cells were incubated with conditioned media of DENV-2 infected cells, in the presence or absence of imatinib. Alternatively, HMEC-1 cells depleted of c-ABL were used as described in previous sections. These experiments were performed in serum-depleted media. These conditions did not trigger motility efficiently. Accordingly, scratched monolayers incubated with control conditioned media displayed almost no motility (less than 1% wound closure, Figure 7A–C, grey). Conversely, cells treated with conditioned media from DENV-2-infected cells (DENV-2-CM and amiScr + DENV-2-CM) showed a slight, but significant, increase in wound healing (Figure 7A–C, black and green). Importantly, increased motility was abrogated by either treatment with imatinib (Figure 7A–C, blue) or prior depletion of c-ABL using microRNA targeting (Figure 7A–C, pink). Together, these data indicate that DENV-2 infection triggers a differentiation process compatible with EndMT characterized by decreased expression of endothelial markers, increased expression of mesenchymal markers, and increased motility.

## 4. Discussion

DENV infection remains a primary health concern worldwide due to its high incidence, rapid global spread due to different reasons including climate change, severe complications, lack of effective treatments against the most acute forms of the disease, etc. Mortality and morbidity are common in the most serious presentation of the disease, severe dengue. Severe dengue emerges from plasma leakage, which is mainly due to endothelial dysfunction. From this, it becomes clear that targeting endothelial targets involved in viral pathogenesis, particularly during the onset of severe dengue, could be a viable strategy to reduce the burden of this common infectious disease.

The cellular (c-) kinase ABL is a cytoplasmic Tyr kinase involved in cell survival and cytoskeletal reorganization. c-ABL is also a crucial player in different viral infectious processes, including entry and receptor binding of polyomavirus [32], Coxsackievirus [33], HIV [34], poxvirus virion release [35], and in post-infective stages of DENV-2 [24]. The data herein demonstrated that c-ABL is not only required for post-infective (viral release) stages, but also for early stages of infection (viral entry). This is likely related to the effect of DENV2 infection on the expression and activity of c-ABL (Figure 1A,B).

Alterations of actin dynamics are typical events associated with DENV and Zika entry and virion release, compromising the barrier function of endothelial cells [36,37,38,39]. Treatment with actin polymerization inhibitors such as cytochalasin D reduced DENV viral entry and inhibited the release of new viral progeny [40]. The role of c-ABL in the regulation of actin dynamics is likely context-dependent. In some models, c-ABL was found to lie upstream of Rac GTPases, and its inhibition prevented actin-based membrane protrusion [18,41]. On the other hand, c-ABL has been decisively shown upstream of RhoA in HGF-treated cells [30]. Due to this divergence, it was necessary to address the role of c-ABL in HMEC-1 cells. The present data indicate that c-ABL inhibition with imatinib decreases the density of actin stress fibers, suggesting that, in these cells, pharmacological targeting of c-ABL seems to inhibit RhoA-dependent phenomena, e.g., actin organization into stress fibers. Of note, stress fibers remained mainly unaffected in c-ABL-depleted cells using plasmid-based targeting. This is because genetic targeting of c-ABL reduced the levels of the protein by 50%, whereas imatinib likely affects 100% of the expressed protein (Figure 3). Strikingly, DENV-2 infection does not alter actin organization significantly (Figure 5) despite having dramatic effects on the expression of endothelial and mesenchymal markers (Figure 5 and Figure 6). This suggests that the effects of DENV-2 infection on endothelial permeability are more dependent on the repression of endothelial junctional molecules rather than an increase in cell contraction, which would cause dramatic modifications to the architecture of the actin cytoskeleton.

These data indicate that DENV-2 infection in HMEC-1 cells increases c-ABL expression and promotes its activation (Figure 1). Due to the critical role of c-ABL in the formation and maintenance of adherens junctions [18], this is in agreement with c-ABL acting as an obligatory mediator of the morphological/transitional events triggered by infection. The levels of phosphorylated CRK-II are dependent on c-ABL activity (Figure 3C). Phospho-CRK-II is enriched in protrusive regions of the cell [42] and promotes Rac-mediated protrusion [43], which may underlie some of the motility effects observed here.

According to the data presented herein, DENV-2 infection triggers events consistent with the onset of endothelial–mesenchymal transition. Indeed, DENV-2-conditioned media triggered a decrease in the levels of endothelial markers involved in cell–cell adhesion such as VE-cadherin and ZO-1, simultaneously triggering expression of mesenchymal markers such as N-cadherin and vimentin. The crucial components present in the conditioned media that induce these responses are currently under investigation in our laboratories. In this regard, it has been shown that viral NS1 increases endothelial permeability and triggers vascular leakage [11]. While NS1 is likely a component of the conditioned media used herein, the presence of other non-structural viral proteins, cytokines, and soluble factors, e.g., TNF-alpha, cannot be ruled out. These factors may have synergic or additive effect to that of NS1 in increasing endothelial permeability and migration. Current research in our laboratories also aims to characterize the specific roles of NS1 in endothelial permeability, with focus on extracellular (secreted) NS1 oligomers and intracellular endothelial interacting partners.

A previous report indicated that DENV induces vascular permeability through phosphorylation and internalization of VE-cadherin in HMEC-1 cells [12]. Another study showed that endothelial cells can co-express N-cadherin and VE-cadherin. Their roles seem to differ, with VE-cadherin (a type II cadherin) mediating endothelial–endothelial interactions and N-cadherin (a type I cadherin) mediating the interaction of endothelial cells with other cell types within their microenvironment, e.g., smooth muscle cells, or pericytes [44]. In addition, a more recent study has shown that N-cadherin can form heterotypic interactions during chain migration of tumor cells dragged by leader fibroblasts [45]. Interestingly, the affinity of homotypic VE-cadherin interactions is higher (5–20-fold) than that of N-cadherin interactions [46]. The acquisition of N-cadherin is thus likely related to the ability of endothelial cells to engage in motile processes that require their interaction with cells in their surroundings, e.g., pericytes [47]. These interactions are more labile, thus easier to break upon application of mechanical force [48]. Therefore, the transition of endothelial cells into a more mesenchymal state weakens homotypic, VE-cadherin-dependent junctions, enabling them to establish more labile interactions with other cell types as they become more motile. In addition, DENV-2 also promotes matrix metalloproteinase 9 (MMP-9) secretion and activation of nuclear factor κB (NF-κB), leading to degradation of tight junction proteins and apoptosis [49,50,51,52]. The data herein demonstrate that these events are linked to phenotypic transitions and are caused by soluble mediators released during the infection process. It is crucial to note that, in the data shown in Figure 5 and Figure 6, the differences in the levels of endothelial and mesenchymal markers examined likely reflect basal changes corresponding to differential, possibly intermediate, EndMT stages of the culture triggered by the exposure to viral or virally induced factors. Recent studies have indicated that EMT/EndMT are not binary states, but there is a continuum of intermediate stages between 100% epithelial/endothelial and 100% mesenchymal [53]. It is worth noting that downregulation of endothelial-specific markers does not necessarily lead to increased endothelial permeability or cell migration. In this regard, ectopic expression of E-cadherin (which is an epithelial marker) in pancreatic cancer cells increased their invasive ability [54], implying that increasing or decreasing individual markers of morphological transitions does not necessarily, in isolation, underlie the phenotypic changes observed in these cells.

**Figure 8:**
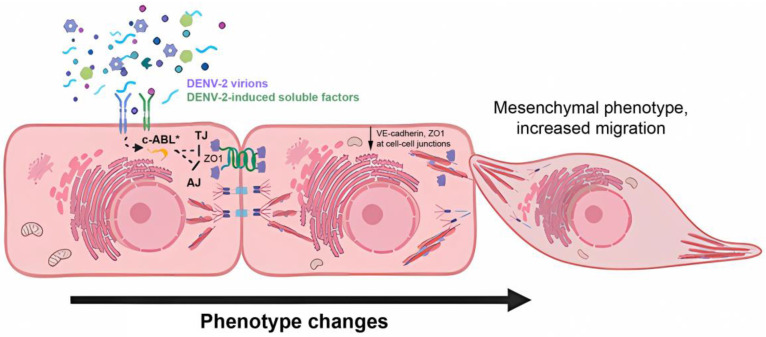
Working model of induction of EndMT in endothelial cells by soluble factors induced by DENV-2 infection. Soluble factors produced during DENV-2 infection, which can be of viral or cellular origin, induce phenotypic changes in endothelial cells that depend on the activation of c-ABL kinase, increasing cell migration. c-ABL*, active c-ABL; TJ, Tight Junction; ZO1, Zonula Occludens-1; AJ, Adherens Junction; VE-cadherin, Vascular-Endothelial-cadherin.

In summary, a picture emerges in which severe dengue is caused by endothelial dysfunction, due to apoptosis [50] and/or repression of mediators of cell–cell junctions [12,49], which appears to be induced by a DENV-2-induced trans-differentiation event in endothelial cells (Figure 8). These results also suggest that repurposing the anti-cancer inhibitor imatinib could be a useful strategy to control severe dengue, by countering the trans-differentiation events triggered by the release of soluble factors associated with DENV infection of endothelial cells.

## Data Availability

Original data are available from the authors upon reasonable request.
