# Correction: Escudero-Flórez et al. Dengue Virus Infection Alters Inter-Endothelial Junctions and Promotes Endothelial–Mesenchymal-Transition-like Changes in Human Microvascular Endothelial Cells. *Viruses* 2023, *15*, 1437

**DOI:** 10.3390/v15112252

**Published:** 2023-11-13

**Authors:** Manuela Escudero-Flórez, David Torres-Hoyos, Yaneth Miranda-Brand, Ryan L. Boudreau, Juan Carlos Gallego-Gómez, Miguel Vicente-Manzanares

**Affiliations:** 1 Molecular and Translation Medicine Group, University of Antioquia, Medellin 050010, Colombia; manuela.escuderof@udea.edu.co (M.E.-F.); david.torresh@udea.edu.co (D.T.-H.); yaneth.miranda@udea.edu.co (Y.M.-B.); 2 Division of Cardiovascular Medicine, Department of Internal Medicine, University of Iowa Carver College of Medicine, Iowa City, IA 52242, USA; ryan-boudreau@uiowa.edu; 3 Molecular Mechanisms Program, Centro de Investigación del Cáncer, Instituto de Biología Molecular y Celular del Cáncer, Consejo Superior de Investigaciones Científicas (CSIC), Universidad de Salamanca, 37007 Salamanca, Spain

## Addition of an Author

**Ryan L. Boudreau** was erroneously not included as an author in the original publication [1]. The corrected Author Contributions statement appears here. The authors state that the scientific conclusions are unaffected. This correction was approved by the Academic Editor. The original publication has also been updated.

## Detailed Contributions

Methodology: Dr. Boudreau designed and generated the mi-cAbl constructs to create a robust model for c-Abl knockdown through gene silencing.

Investigation: Dr. Boudreau cloned and screened a panel of four mi-cAbl vectors to determine which was best for knockdown (qRT-PCR and Western blots resulting in effective gene silencing as demonstrated throughout the manuscript). 

Resources: mi-cAbl vectors served as a crucial custom resource made for and enabling these studies.

Writing—Review and Editing: Dr. Boudreau reviewed the manuscript and made recommendations for citing the appropriate literature on artificial miR designs.
